# Median raphe cyst of the penis: a case report and review of the literature

**DOI:** 10.1186/s13256-019-2133-5

**Published:** 2019-07-14

**Authors:** M. M. Aarif Syed, Bibush Amatya, Seema Sitaula

**Affiliations:** Department of Dermatology and Venereology, Institute of Medicine, Tribhuvan University, Maharajgunj, Kathmandu, Nepal

**Keywords:** Median raphe cyst, Penis, Scrotum, Perineum, Excision

## Abstract

**Background:**

A defect in embryological development or closure of median raphe may lead to formation of cyst(s) anywhere in the midline from glans to anus. These cysts are referred to as median raphe cysts, an uncommonly encountered clinical condition. The cyst is generally solitary, with the penile shaft being the most common location, with average size of around 1 cm. The diagnosis is mostly clinical and confirmed histologically. We report a case of a patient with a rare histological variant of median raphe cyst and provide a focused review on presentation, histopathology, and management.

**Case presentation:**

A 29-year-old unmarried Nepali man presented to our clinic with an asymptomatic, solitary, soft, translucent, nontender cystic lesion of about 1-cm diameter at the ventral aspect of glans penis, close to the meatus, that had been noticed at the age of 3 and was nonprogressive for the past 15 years. Ultrasonography demonstrated an isoechoic cystic lesion at the tip of the penis, separated from the urethra, and lying entirely within the mucosa without any evidence of solid component, septation, or vascularity. On the basis of clinical and ultrasonographic findings, a diagnosis of median raphe cyst of the penis was made. The cyst was excised with the patient under local anesthesia, and there was no evidence of recurrence in 2 years of follow-up. The histopathological examination with Hematoxylin and eosin staining showed the cyst wall was lined partly by ciliated pseudostratified columnar epithelium and partly by columnar epithelium with apical mucin.

**Conclusions:**

Median raphe cyst is an uncommon, mostly asymptomatic condition in young patients. The cyst may occur anywhere along the midline from glans to anus. The diagnosis is clinical with histological confirmation. Excision is the treatment of choice with minimal chance of recurrence.

## Background

The male external genitalia develops from the genital tubercle, which contains two urethral folds and the scrotal swellings that fuse in the midline to form the scrotum. The center of fusion is marked by the penile, scrotal, and perineal raphe. A defect in embryological development or closure of the median raphe may lead to formation of cyst(s) in the midline, and hence the name *median raphe cyst* (MRC). However, there are other theories also explaining the pathogenesis [1]. Mermet first described the condition in 1895 [2]. The location of the cyst varies anywhere in the midline from glans to anus [3–5]. MRC is an uncommon benign lesion, mostly presenting to surgeons, urologists, or pediatricians and sometimes to dermatologists [5–7]. Hence, it becomes imperative for dermatologists to know this condition and differentiate it from other lesions of penis, scrotum, or perineum. We report a case of a patient with a rare histological variant of MRC. We also review the clinical presentation, pathogenesis, histopathology, investigations, complications, and management of MRC of the penis. The primary databases searched for the review were PubMed, Google Scholar, the Cochrane Library, and Hinari. The search terms included “median raphe cyst,” “genitoperineal raphe cyst,” “mucoid cyst of penis,” “apocrine cystadenoma of penis”, “hydrocystoma of penis,” “epidermoid cyst of penis,” and “parameatal cyst.”

## Case presentation

A 29-year-old unmarried Nepali man presented to our clinic with a complaint of soft, painless swelling at the tip of the penis since childhood. His mother had noticed the swelling when the patient was at the age of 3, and the swelling had increased in size for a few years to reach its present dimension. However, the swelling had been nonprogressive for the past 15 years. He had no complaints of pain, itching, burning, tenderness, discharge, trauma, or oozing. The lesion did not interfere with urination or penile erection. He did not have any history of congenital anomaly, any medical illness, or similar lesions in family members. The patient’s only concern was cosmetic and the possibility of interference in sexual activity, because he was planning to get married soon.

**Fig. 1:**
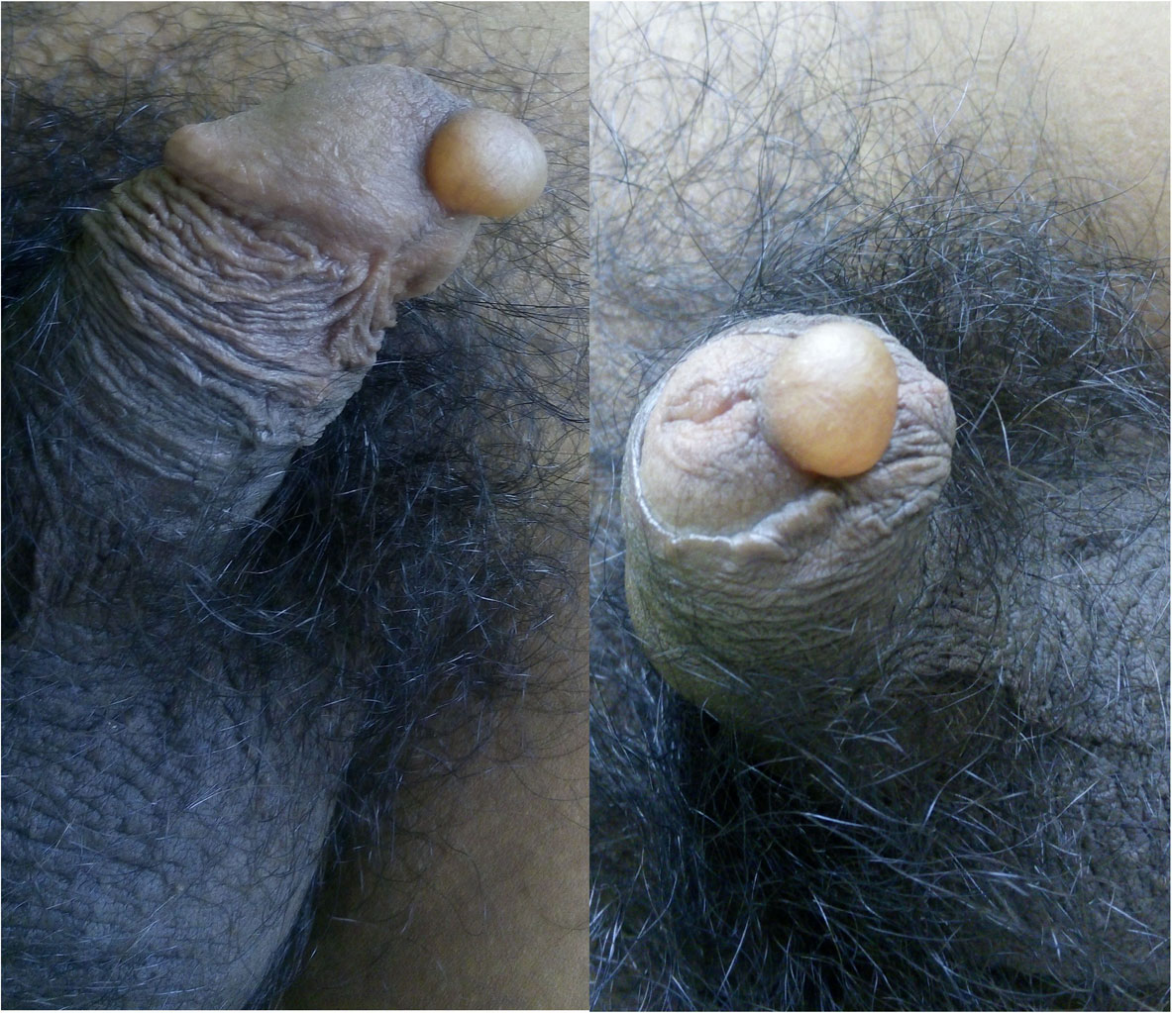
A solitary, soft, translucent cystic lesion of about 1-cm diameter seen at the ventral aspect of glans penis, close to the meatus

**Fig. 2:**
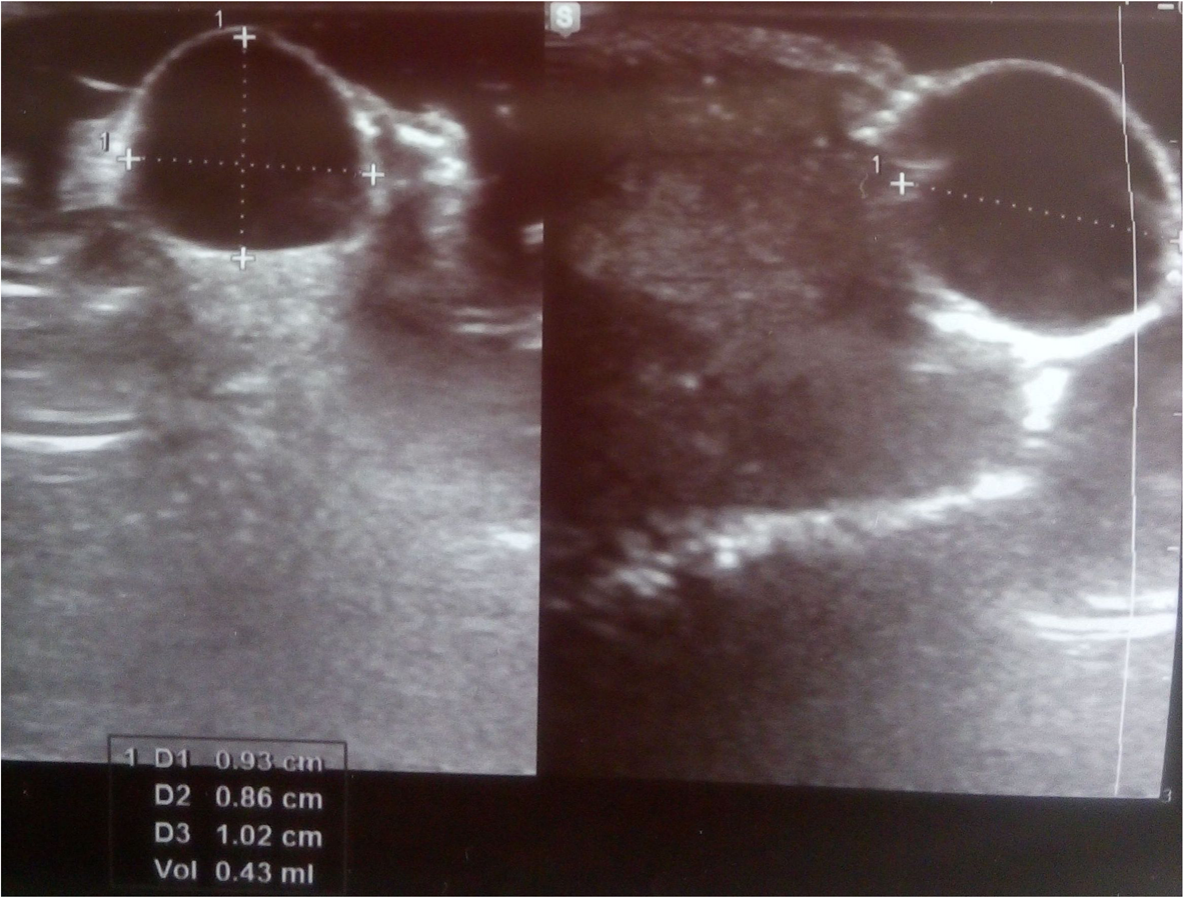
Ultrasonography of the cyst showing a well-circumscribed isoechoic lesion without any extension to the urethra

His physical examination revealed a solitary, soft, translucent cystic lesion of about 2-cm diameter at the ventral aspect of the glans penis, close to the meatus, but not involving the margins of the urethral orifice (Fig. 1). The overlying mucosa was shiny, whereas the surrounding mucosa was normal. The cyst  was soft and nontender on palpation. The urethral opening was not obstructed, and examination of other regions of the penis, scrotum, and perineal region were unremarkable. No palpable inguinal lymphadenopathy was seen. The results of the patient’s urinalysis and hemogram were normal. Ultrasonography (USG) of the cyst was advised, which demonstrated an isoechoic cystic lesion at the tip of the penis (Fig. 2). There was no evidence of a solid component, septation, or vascularity within the cyst seen by USG (Fig. 2). The urethra was separated from the cyst, which was entirely within the mucosa (Fig. 2). On the basis of clinical and USG findings, a diagnosis of MRC of the penis was made.

**Fig. 3:**
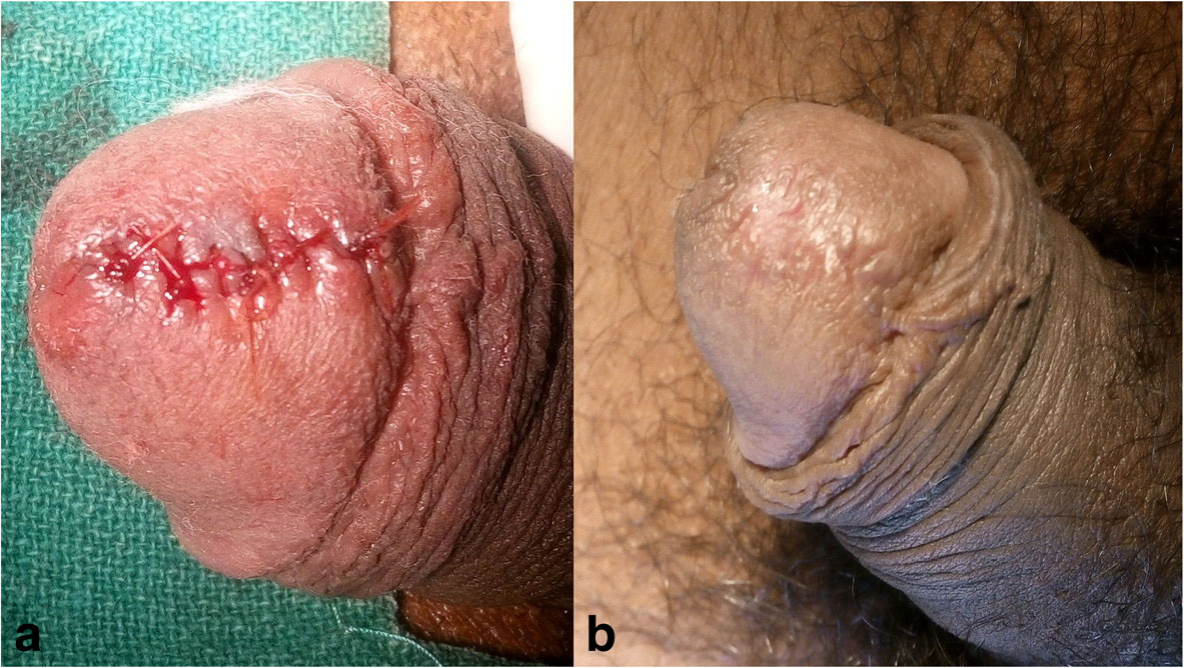
**a** and **b** Glans penis immediately following excision of the cyst and after healing, leaving no residual effect

**Fig. 4:**
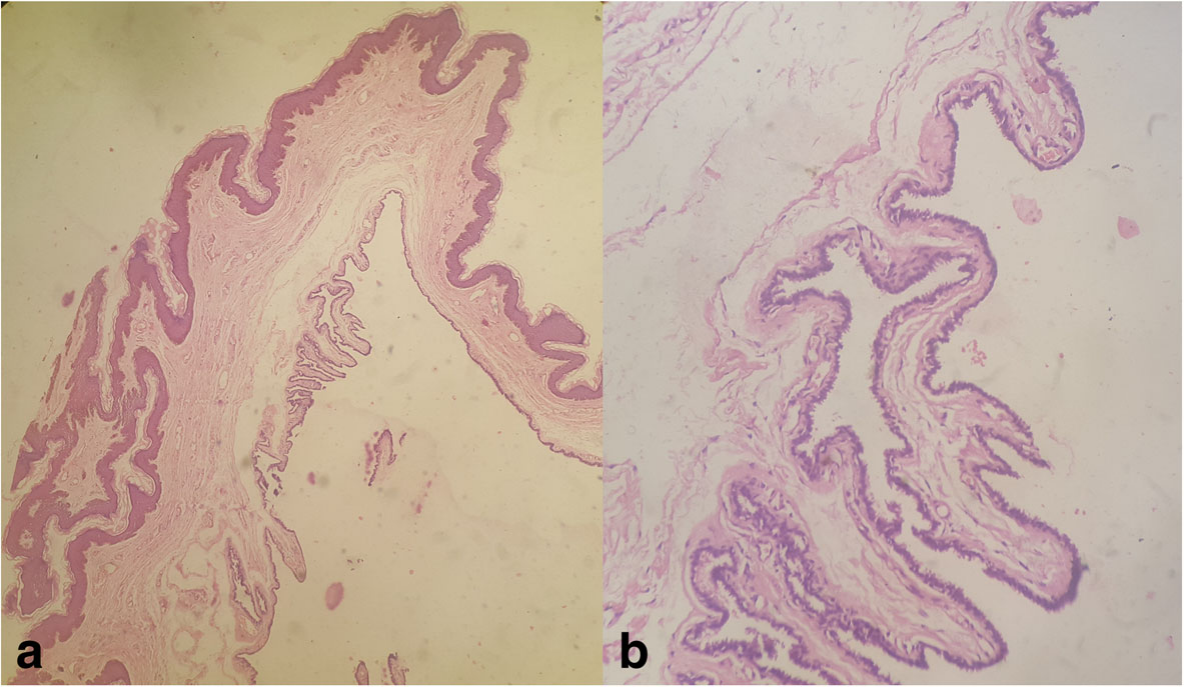
**a** Hematoxylin and eosin (H&E) stain (original magnification ×200) showing cystic cavity lined externally by normal skin and internally by pseudostratified columnar epithelium. **b** H&E stain (original magnification ×400) showing cyst wall lined partly by ciliated pseudostratified columnar epithelium and partly by columnar epithelium

The cyst was excised with the patient under local anesthesia (Fig. 3a, b). During excision, the cyst ruptured, releasing the mucinous content. The whole specimen was sent for histopathological examination. Hematoxylin and eosin (H&E) staining showed the cyst wall was lined partly by ciliated, pseudostratified columnar epithelium and partly by columnar epithelium with apical mucin (Fig. 4a, b). The lamina propria showed mild chronic inflammatory infiltrates. No features of dysplasia or malignancy were noted. IHC could not be done, owing to technical and financial reasons. The patient was followed for 1 year. The site of the excision healed without any residual effect (Fig. 3b). There were no issues related to urination or sexual activities. Recurrence was not observed during the 2-year follow-up period.

## Discussion and review

### History

MRC is a rare congenital entity. Earlier literature consisting of case reports are mainly from Japan [1, 8–11]. However, Asarch *et al.* (USA) compiled six cases reported as early as 1979 [7]. Nagore *et al.* described a case series of five patients from Spain in 1998 [6]. In recent decades, cases have been reported from other parts of the world as well (also see Table 1).

**Table 1:** Reports with two or more than two cases of median raphe cyst

Authors	Year	No. of cases	Location
Asarch *et al.* [7]	1979	6	USA
Otsuka *et al*. [11] (genitoperineal raphe)	1990	160	Japan
Little *et al*. [12]	1992	2	USA
Pellicé i Vilalta and Luelmo i Aguilar [13]	1997	2	Spain
Nagore *et al*. [6]	1998	5	Spain
Otsuka *et al*. [1]	1998	3	Japan
López-Candel *et al.* [14]	2000	2	Spain
Dini *et al*. [15]	2001	2	Italy
Navarro *et al*. [16]	2009	2	Spain
Verma [17]	2009	2	India
Shao *et al*. [5]	2012	55	Taiwan
Matsuyama *et al*. [3]	2016	23	Japan
Navalon-Monllor *et al*. [4]	2017	28	Spain
Kumar *et al*. [18]	2017	2	India

Several terms have been used in the past to describe the condition. It includes genitoperineal cyst of median raphe [19, 20], mucoid cyst of penis [21], apocrine cystadenoma/hydrocystoma of penis [22, 23] and epidermoid cysts [24]. A cyst close to the meatus has been referred to as parameatal cyst [1, 25, 26]. However, in the view of authors, the term that could be synonymous to MRC, is genitoperineal cyst of median raphe. It’s still debatable if all these entities are actually same or different [15]. One fact that unites these conditions is that they all reflect developmental defect in male genitalia during embryogenesis. Mucoid cyst may result from faulty closure of penoscrotoperineal raphe with sequestration of ectopic urethral mucosa [21]. An apocrine cystadenoma/hydrocystoma has bluish hue on clinical examination, while pathological details would reveal focal areas of decapitation secretion in epithelial lining, along with a myoepithelial layer [22, 23]. An epidermoid cyst may result due abnormal closure of the median raphe during embryogenesis or, rarely, following mechanical implantation in acquired cases [27]. A parameatal cyst may arise due to obstruction of paraurethral duct or anomalous fusion of urethra [28].

### Clinical presentation

The cyst is most commonly noticed in the first decade of life; however, because of the condition being asymptomatic, the patient often presents during second to third decades of life. Our patient’s mother first noticed the cyst when he was at the age of 3, but the patient came for medical advice at the age of 29 years, when he was planning to get married. Shao *et al.* mentioned the mean age of presentation to be 26.7 years with a bimodal age distribution at approximately 1–10 years and 21–40 years [5]. Navalón-Monllor *et al.* found a slightly lower mean age (24.6 years) with similar bimodal age characteristics, having higher presentation during the first and third decades of life [4] (also see Table 2).

**Table 2:** Individual cases of median raphe cyst reported with age < 1 year and > 60 years

Authors	No. of cases	Age of presentation
Verma [17]	2	3 months and 6 months
Shibagaki *et al*. [9]	1	4 months
Wang *et al*. [20]	1	4 months
Park *et al*. [29]	1	8 months
Kumar *et al*. [18]	1	9 months
Scelwyn [30]	1	62 years
Sagar *et al*. [31]	1	65 years
Dini *et al*. [15]	1	67 years
Navarro *et al*. [16]	1	68 years
Bhasin *et al*. [32]	1	76 years

Our patient was asymptomatic, and the cyst had ceased to grow for more than a decade. MRCs are mostly asymptomatic [3–5, 7, 31] and grow proportionately with body size [33]. Though asymptomatic, MRC has the potential to cause psychological embarrassment and discomfort [31]. Parameatal cysts are more likely to give rise to symptoms of urinary obstruction [4, 5, 34]. These symptoms are limited to dysuria, urinary frequency, or deflected urinary stream [3, 16, 35]. Pain, if present, is an indication of infection [4, 5, 12]. Rarely, hematuria, hematospermia [5], and difficulty in sexual function [34, 36] may be presenting complaints. A cyst near the anal region is mistaken for hemorrhoids [37]. The most common reason for consultation is aesthetic [4, 15]. The more distal the cyst and earlier the age at presentation, the greater the chances of it being symptomatic [5]. A case of appearance of MRC in the shaft of the penis following intense, prolonged sexual intercourse has been described [38].

The cyst is generally solitary [3–5], sometimes double [3], and rarely presents as a chain of cystic swellings along the median raphe [4, 10, 33, 39]. The chain of cysts gives a cordlike appearance and has been described as canaliform MRCs [17, 29, 40]. Another variant is the presence of cysts within the raphe canal, which is an elongated tract along the median raphe [41–44]. Coexistence of a cystic-type lesion and canaliform variant in different regions has also been reported [37]. Videodermatoscopy can help confirm the presence of a true canal [43]. The cyst is always obvious on the surface; however, a case of MRC was reported from the United Kingdom in which MRC presented as nonvisible palpable swelling that was confirmed on USG and magnetic resonance imaging (MRI) [45]. Another variation in presentation could be the beginning of the condition with a single lesion with subsequent appearance of more cysts [46]. The cyst very rarely may have central umbilication, giving rise to differential diagnosis of molluscum contagiosum [47].

The cysts are generally translucent, and the penile shaft is the most common location [3–5]. However, many authors have separately defined parameatal urethral cysts, whose numbers exceed those of cysts in any other location [3, 28, 48, 49]. Consideration of parameatal cyst as a separate condition or a form of MRC needs more discussion in the scientific community. We tend to agree making parameatal the most common variant of MRC. The cyst may have a bluish hue [6], which is more a characteristic of cystadenoma/hydrocystoma of the penis. Pigmented cysts appearing as brown-black in color due to presence of melanocytes and melanin pigment in the epithelial lining have also been reported [10, 46, 50]. Multiple areas are rarely involved. The majority of the cysts do not reach size in excess of 1 cm [4, 5]. However, Matsuyama *et al.* [3] reported that about 70% of the patients in their study had size less than 0.5 cm. Scrotal cysts tend to be larger than cysts elsewhere [5]. In our patient, the size of the cyst was about 1 cm, which made it larger than the mean observed size of these cysts. MRCs with sizes in excess of 2 cm have also been reported [16, 32, 50]. A comparative analysis of a few clinical features of MRC as reported in three large reviews is presented in Table 3. MRC has to be differentiated from a number of conditions. A differential diagnosis by location is given in Table 4.

**Table 3:** Clinical characteristics of median raphe cyst in three large reviews

	Shao *et al*. [5]	Matsuyama *et al*. [3]	Navalon-Monllor *et al*. [4]
No. of cases	55^a^	23	28
Cyst size range	0.2–2.1 cm	0.1 to > 1 cm	0.5–3.5 cm
Mean size of cyst	0.88 cm	NA	1.1 cm
Cyst location			
Parameatal	19 (33.9)	^b^	8 (28)
Glans penis	4 (7.1)	–	–
Penile shaft	24 (42.9)	11 (47.8)	10 (36)
Scrotum/perineum	2 (3.6)	2 (8.7)	2 (7)
Prepuce	7 (12.7)	–	3 (11)
Multiple areas		4 (17.4)	2 (7)
Corona/balanic frenulum		6 (26.1)	3 (11)
Symptoms			
Asymptomatic	40 (72.7)	19 (82.6)	22 (79)
Symptomatic	15 (27.3)	4 (17.3)	4 (21)

*MRC* Median raphe cyst, *NA* Not available

Data in parentheses are percentages

^a^55 Patients with 56 MRCs

^b^Authors have taken parameatal urethral cysts (PUCs) as a separate category and compared them with MRCs. The PUC group had 46 patients, and the MRC group had 23 patients. Thus, PUCs formed in 66% of their cases

**Table 4:** Differential diagnosis of median raphe cyst by location

Location	Differential diagnosis
Glans penis	Urethral diverticulum, capillary/cavernous hemangioma, glomus tumor, leiomyoma [51]
Shaft	Steatocystoma, molluscum contagiosum, trichilemmal cyst [52], dermoid and epidermoid cysts, lipoma
Scrotum	Steatocystoma, calcinosis cutis
Perineum	Cowper gland cyst, lipoma, epidermoid cyst
Perianal	Hemorrhoids, perianal polyp, pilonidal cyst, hidradenitis, teratomas [53]

### Pathogenesis

A debate on the pathogenesis of MRC has also seen varied views. The cyst may represent an embryological developmental anomaly of male genitalia or a defect in closure of the median raphe. The genital tubercle, two urethral folds and the scrotal swellings, give rise to male external genitalia. The scrotal swellings fuse in the midline to form the scrotum, which leaves a permanent surface marking in the form of median raphe. An incomplete closure of the genital or urethral fold gives rise to epithelial rest. The rest may develop into either a cyst or a canal, depending on the presence or absence of an opening on skin surface [54]. A cyst can appear even after primary closure from split-off outgrowths of embryonic epithelium [54]. A theory that also finds support is “tissue trapping,” in which epithelial rests may get buried during midline fusion and evolve into a cyst or canal [12]. Autologous transplant of skin tissue specimens has shown the development of cysts at the transplant sites, lending credibility to the tissue-trapping theory [55]. Shiraki, on the basis of a study of nine cases of parameatal cyst of glans penis, proposed the occlusion of paraurethral ducts resulting in cyst formation as an explanation [48]. A congenital obliteration of these ducts hampered the physiological drainage and led to development of cysts [48]. Infection and trauma are acquired contributory factors in the obstruction [49]. Light and electron microscopic findings of Otsuka *et al.* support this theory [1]. Cole and Helwig proposed an alternative, stating that these cysts could be the result of sequestered ectopic periurethral glands of Littre [21]. However, strong evidence in support of this explanation is still lacking.

### Investigations

The diagnosis is mostly clinical and is confirmed histologically. As in our patient, USG shows an isoechoic cystic lesion [34, 45]. It can help to rule out vascularity and continuity to overlying or underlying structures. However, it has a very limited role in diagnosis and is not frequently ordered. The use of MRI is also not encouraged and reveals low T2-weighted signal of a soft tissue lesion without any appreciable contrast enhancement [45]. Like USG, MRI also helps in determining the anatomical extent of the cyst [16]. A urethrogram will not show any communication between the cyst and urethra [8].

### Histopathology/IHC

The cyst in our patient was unilocular, which is the condition most frequently encountered. However, multilocular cysts can also be seen [16]. The pathogenetic mechanism and type of tissue trapped explain the tissue lining [6, 7]. Trapping of the proximal and distal urethra would result in pseudostratified and stratified squamous epithelial lining, respectively. If the periurethral glands are trapped, they will form a glandular lining of cyst. Shoa *et al.* published an extensive report based on histopathological findings in 55 cases of MRCs [5]. They classified MRCs into four groups, depending on the type of epithelial lining of the cyst wall:

1. Urethral: Lined by pseudostratified columnar epithelium, such as the urothelium
2. Epidermoid: Lined by squamous stratified epithelium
3. Glandular: Lined by urethral epithelium with interspersed glandular structure
4. Mixed: Lined by more than one type of epithelium, such as urethral epithelium with squamous metaplasia or mucinous cells, or all the three coexisting

We would like to extend this classification and add two more categories: ciliated and pigmented. The ciliated type is characterized by the presence of ciliated cells interspersed with pseudostratified [16, 26, 47, 56] or columnar [31, 57, 58] epithelium. The pigmented variant appears brown-black in color due to the presence of melanocytes and melanin pigment in the epithelial lining [10, 46, 50]. Pigment granules can become evident with Fontana-Masson stain [50]. Though case reports on these two groups always claimed them to be rare findings, we would like to challenge this claim. Ciliated cysts have been reported more frequently than glandular variant (see Table 4). The epidermoid type, too, is uncommonly reported (see Table 4) [5], but it has never been referred to as rare. Some authors attribute ciliated cysts to an additional abnormality of embryological development [47], whereas others refer to it as a result of metaplastic changes in the urothelium [58]. In our extensive literature search, we could only find three cases of pigmented variant [10, 46, 50], which in our view makes them truly rare.

Unal *et al.* [56], in their compilation of cases of ciliated cysts, mentioned six cases, including their own, as documented in the literature. Perhaps they missed the case reported by Navarro *et al*. in 2009 [16] and the one reported by Amaranathan *et al*. in 2013 [57] (also see Table 4). We extend this list and include these two cases. We further include our patient’s case as well, which also showed ciliated, pseudostratified epithelium, thus taking the number of total reported cases to nine. In Table 5, we compile the histopathological findings of 2 large reviews of MRC and 29 individual case reports/series documented separately as one group.

**Table 5:** Histopathological findings in 2 large reviews and a third group comprising 29 individual case reports

	Shao *et al*. [5]	Navalon-Monllor *et al.* [4]	Third group
Number of cases	56^a^	28	29
Type of epithelium			
Urethral	31 (55.4)	15 (54)	[37, 44] [6]^b^X5 [7]^b^X5 Total: 12/29 (41.3)
Epidermoid	3 (5.4)	2 (7)	[29, 33, 42] Total: 3/29 (10.3)
Glandular	2 (3.4)		[1]^c^X3 Total: 3/29 (10.3)
Mixed	20 (35.7)	11 (39)	[7, 15, 20, 34, 40, 45, 59–61] [18]^d^X2 Total: 11/29 (37.9)
Rare variants			
Ciliated	–	–	[16, 30, 31, 47, 56–58, 62] + our patient
Pigmented	–	–	[10, 46, 50]

Data in parentheses are percentages

X: signify that this particular reference had reported that many number of MRC

^a^55 Patients with 56 median raphe cysts

^b^Five cases

^c^Three cases

^d^Two cases

IHC has been undertaken in several case reports (Table 6). The most consistent staining is seen with cytokeratin 7, strongly indicative of urethral origin of the cyst (Table 6). Positive cytokeratin 13 lends further support. Carcinoembryonic antigen positivity has also been regularly reported, except by Persec *et al.* [60]. Cytokeratin 20 and smooth muscle actin results remain negative.

**Table 6:** IHC results in various case reports

	Dini *et al*. (2001) [15]	Cardoso *et al*. (2005) [61]	Sagar *et al*. (2006) [31]	Koga *et al*. (2007) [62]	Persec *et al*. (2013) [60]	Deliktas *et al*. (2015) [34]	Çalışkan *et al*. (2015) [59]
CK7	+	+	+	+	+	+	+
CK13	+	+					
CK20	–	–	–	–	–	–	–
CEA	+	+	+	+	–	+	+
Anti-S100		–	+	–	–		+
SMA			–	–	–		–
Desmin			–				
EMA		+	+	+	+		
HMFG-1		–					
CAM5.2	+						
GCDFP-15				–			+

*Abbreviations: CEA* Carcinoembryonic antigen, *CK* Cytokeratin, *EMA* Epithelial membrane antigen, *HMFG* Human milk fat globulin, *SMA* Smooth muscle actin, *GCDFP-15* Gross cystic disease fluid protein-15

### Complications

The cyst may be secondarily infected and manifest as pain, tenderness, erythema, or pus discharge. About 16% of patients reported by Shao *et al.* had infected cysts [5]. The most common organism found is *Neisseria gonorrhoeae* [63, 64]. Infection with *Trichomonas vaginalis* can also occur, but it is rare [65]. However, these are case reports of infection of the median raphe rather MRC. *Staphylococcus aureus* infecting MRC of the scrotum and penis has been noted in old case reports [54, 66]. Infection of MRC has been uncommonly documented in recent decades. The culture of the cystic content can help confirm the infective organism. Infection is generally a phenomenon noticed after sexual intercourse. The cyst may also be traumatized by other means and become infected. Urinary obstruction, if due to a parameatal cyst, does not lead to urethritis. MRCs never communicate with the urethra; however, recently, a case of an epidermoid variant traversing the corpus cavernosum has been reported [67]. Another case of MRC in the scrotum was found to mimic a serous tumor and was associated with cryptorchidism [68]. A few very unusual cases of MRC reported in the literature are compiled in Table 7.

**Table 7:** A few very unusual cases of median raphe cyst reported in the literature

Authors	Title	Reference. no.
Bhasin *et al*.	Giant median raphe prepuceal cyst in an elderly male.	[32]
Takahashi *et al.*	Congenital median raphe cysts: coexistence of cystic lesions and canal-like lesions.	[37]
Sharkey *et al*.	Postcoital appearance of a median raphe cyst.	[38]
Yu *et al.*	A case of epidermoid median raphe cyst traversing the corpora cavernosa.	[67]
Hara *et al*.	Median raphe cyst in the scrotum, mimicking a serous borderline tumor, associated with cryptorchidism after orchiopexy.	[68]

### Treatment

Spontaneous resolution has been reported [3, 9, 28]. Observation is another option when the cyst is small and the child is asymptomatic [12]. Because the cyst mostly remains symptom-free, some patients deny any active treatment [6]. If left untreated, the cyst may rupture on its own and heal uneventfully [12]. Aspiration of the cyst is associated with recurrence [5, 33, 48]. Marsupialization or unroofing is not recommended, because it may lead to gaping sinus [48]. However, marsupialization has been found to be effective in treating canals [41]. Median raphe canals have also been treated with incision followed by electrodessication [7]. Excision followed by primary closure remains the treatment of choice [3–5, 7] and provides cosmetically acceptable results. When the cyst lies in the prepuce, circumcision can also be performed [32, 57]. A giant cyst hanging at the frenulum has been treated with excision and repair by frenuloplasty [49].

Excision is associated with excellent results, with no evidence of recurrence in 6 months [36], 1 year [34, 45], and 4 years [56] of follow-up. In a case series with six patients, Asarch *et al.* noted recurrence in one of the patients after 5 years, and the cyst was reexcised with no subsequent recurrence [7]. One of the patients of Shao *et al*. developed a fistula following excision [5]. In a retrospective study spanning 14 years, Matsuyama *et al*. did not identify a single case of recurrence following treatment [3].

## Conclusions

MRC is an uncommon, mostly asymptomatic condition seen in young patients. The cyst may occur anywhere along the midline from the glans to the anus. The diagnosis is clinical with histological confirmation. Excision is the treatment of choice with minimal chance of recurrence.

## Data Availability

Data sharing is not applicable to this article, because no data were generated or analyzed during the study. All data (clinical and histopathological) gathered for this study are included in this published article.
